# Presynaptic Inputs to Any CNS Projection Neuron Identified by Dual Recombinant Virus Infection

**DOI:** 10.1371/journal.pone.0140681

**Published:** 2015-10-15

**Authors:** João M. Bráz, Fan Wang, Allan I. Basbaum

**Affiliations:** 1 Department of Anatomy, University of California San Francisco, San Francisco, CA, 94143, United States of America; 2 Department of Cell Biology, Duke University, Durham, NC, 27710, United States of America; University of Kentucky Medical Center, UNITED STATES

## Abstract

Although neuroanatomical tracing studies have defined the origin and targets of major projection neurons (PN) of the central nervous system (CNS), there is much less information about the circuits that influence these neurons. Recently, genetic approaches that use Cre recombinase-dependent viral vectors have greatly facilitated such circuit analysis, but these tracing approaches are limited by the availability of Cre-expressing mouse lines and the difficulty in restricting Cre expression to discrete regions of the CNS. Here, we illustrate an alternative approach to drive Cre expression specifically in defined subsets of CNS projection neurons, so as to map both direct and indirect presynaptic inputs to these cells. The method involves a combination of Cre-dependent transneuronal viral tracers that can be used in the adult and that does not require genetically modified mice. To trigger Cre-expression we inject a Cre-expressing adenovirus that is retrogradely transported to the projection neurons of interest. The region containing the retrogradely labeled projection neurons is next injected with Cre-dependent pseudorabies or rabies vectors, which results in labeling of poly- and monosynaptic neuronal inputs, respectively. In proof-of-concept experiments, we used this novel tracing system to study the circuits that engage projection neurons of the superficial dorsal horn of the spinal cord and trigeminal nucleus caudalis, neurons of the parabrachial nucleus of the dorsolateral pons that project to the amygdala and cortically-projecting neurons of the lateral geniculate nucleus. Importantly, because this dual viral tracing method does not require genetically derived Cre-expressing mouse lines, inputs to almost any projection system can be studied and the analysis can be performed in larger animals, such as the rat.

## Introduction

Traditional neuroanatomical tract tracing technologies do not allow for detailed analysis of the circuits engaged by and with labeled neurons. For example, injection of a retrograde tracer, such as Fluorogold, into the central nervous system (CNS) will inevitably result in tracer uptake by terminals that target interneurons as well as projection neurons at the injection site, making it impossible to conclude that any retrogradely labeled neuron, in fact, targets a particular population of neurons at the injection site. Advances in virology and genetic technologies now make it possible to assess directly the inputs to a neurochemically distinct group of neurons. For example, using the Cre-dependent recombinant pseudorabies virus, BA2001 [1], we showed that serotonergic neurons of the medullary raphe receive direct as well as indirect inputs from a wide variety of brainstem neurons [2]. A variation of this approach uses modified rabies viruses that limit retrograde transport to the immediate presynaptic neuron that targets the Cre-expressing neurons [3].

Despite tremendous advantages, these viral tracing methods are nevertheless limited by the availability of Cre-expressing mouse lines. To overcome this limitation, here we describe a simple, but powerful dual viral injection strategy that does not require pre-existing Cre lines. Instead, we introduce the Cre exogenously. The approach involves injection of retrogradely transported viral vectors that can target Cre expression to defined projection neurons (PN) populations, in combination with a second Cre-dependent, retrogradely transported viral vector (BA2001 or rabies). We demonstrate that this procedure is applicable in any CNS projection neuron population, provided that the PN population can be retrogradely labeled selectively. A particularly powerful advantage of the new approach is that it can be performed in rats as well as mice, and likely in any animal that can transport these viral vectors. By comparing patterns obtained with BA2001 with those obtained with rabies, it is also possible to distinguish between monosynaptic and polysynaptic inputs to particular subpopulations of PNs. In the present report we demonstrate utility and feasibility of the system by examining well-established circuits of the rodent CNS.

## Material and Methods

### Animals

All experiments were reviewed and approved by the Institutional Care and Animal Use Committee at the University of California San Francisco. All viral injections were performed in adult male C57BL/6 mice or adult male Sprague-Dawley rats or in transgenic mice that Cre-dependently express the avian receptor TVA and the G viral protein (Jackson Lab. Maine, USA).

### Viruses

For the dual infections, we used the following viral vectors: a replication incompetent Cre-expressing adenovirus (Ad-Cre; 10^9^ plaque forming units (pfu)/ml); a replication incompetent adenovirus expressing both red fluorescent protein (RFP) and a codon optimized Cre (iCre) under the CMV promoter (Vector Biolabs, USA); a replication incompetent and Cre-dependent BA2001 virus. BA2001 is a thymidine kinase-deficient pseudorabies virus recombinant [1]. Cre-excision restores thymidine kinase expression, which is required for replication, and tau-GFP, a green fluorescent protein axon reporter. In other experiments, we used an AAV vector that Cre-dependently expresses the avian receptor, TVA, and the viral glycoprotein G (AAV-TVA-G; 10^10^ pfu/ml; U. North Carolina Vector Core), generated from the plasmid pAAV-EF1a-FLEX-GTB (Addgene, USA). Finally, we used an RFP-expressing rabies viral system ([3]; Salk Institute, USA) as well as a GFP-expressing rabies viral vector. These rabies viruses only infect and exit cells that express both TVA and G.

### Viral Injections

Injections were performed as previously described [2]. Briefly, animals were anesthetized with ketamine (60 mg/kg, ip for mice and 100 mg/kg, ip for rats) and xylazine (8 mg/kg, ip for mice and 10 mg/kg, ip for rats) and then placed in a stereotaxic apparatus. Following incision of the skin overlying the skull, we made two small burr holes over the regions of interest, e.g., amgydala (Amg) and parabrachial nucleus (PB). We made single injections of 0.5 to 1.0μl (~10^6^ total pfu) of the concentrated viral suspensions in each area. In the case of dual injections in the PB and trigeminal nucleus caudalis (TNC), we exposed the TNC after the Ad-Cre injection into the PB. We made several injections of the viruses (100 nl each; 10^6^ total pfu; BA2001 or AAV-TVA-G) so as to cover the rostral caudal extent of the TNC. Next the skin was sutured, after which the animal was kept under a warming lamp until it recovered from anesthesia, and then returned to standard housing. We previously demonstrated that 24h after injection of BA2001, both mono- and polysynaptic inputs can be detected, presumably due to the rapid transneuronal transfer of BA2001 [2]. Furthermore, we previously showed that there was a significant increase in the number of GFP+ neurons 48h post-injection and a significant decrease at 5 days. For this reason, most animals in the present study were analyzed 2 days after Ad-Cre/BA2001 injection. For the studies of monosynaptic connections using the rabies system, two weeks after the Ad-Cre and/or AAV-TVA-G dual infection, mice received a suspension of rabies at the AAV injection sites. We did not observe morbidity or mortality among the animals infected with BA2001 or rabies.

### Antibodies and Immunohistochemistry

All experiments were performed as previously described [4]. A minimum of 5 animals has been analyzed for each combination of vectors. We used the following antibodies: rabbit anti-GFP (1:2000, Molecular Probe), chicken anti-GFP (1:2000, Abcam), rabbit anti-RFP (1:1000, Clontech), rabbit anti-5HT (1:10000, Incstar), mouse anti-TH (1:2000, RBI), mouse anti-NF200 (1:10000, Sigma), rabbit anti-ßgal (1:10000, CAPPEL), rabbit anti-Pax2 (1:4000, Abcam) and guinea-pig anti-PKCγ (1:7000, Strategic Biosolutions).

## Results

**Fig 1:**
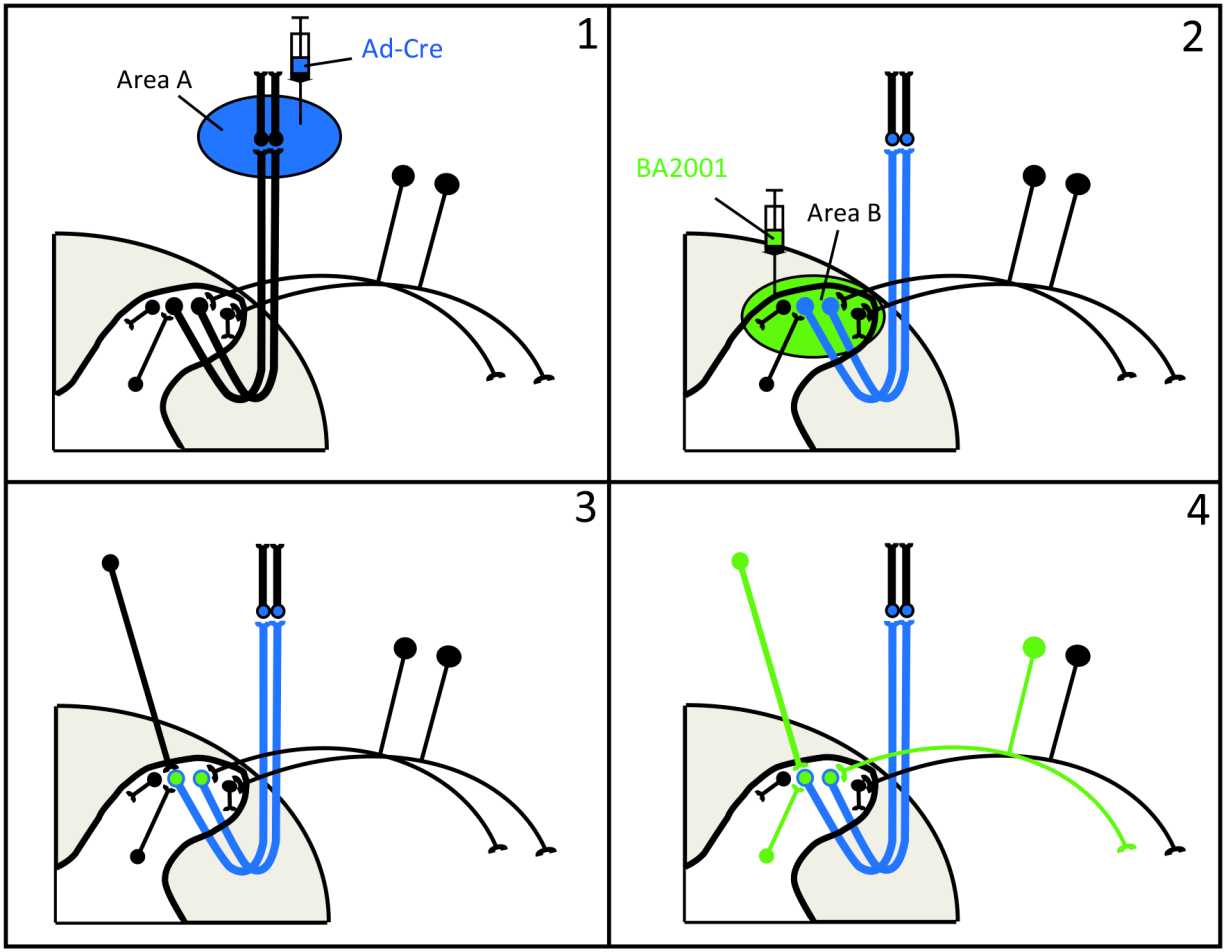
Dual viral infection strategy for the study of presynaptic inputs to subpopulations of spinal cord projection neurons. (1) Injection (blue) of an adenoviral vector expressing Cre (Ad-Cre) in area A of the CNS (e.g. the parabrachial nucleus, thalamus, cervical spinal cord, etc.) results in uptake of the Ad-Cre by spinal cord neurons that project to area A. (2) This initiates Cre expression in all infected projection neurons (Ad-Cre-infected neurons become blue). Subsequent injection of BA2001 (green) in area B of the spinal cord infects all neurons present at the injection site. (3) BA2001 only replicates in projection neurons that express Cre recombinase (blue neurons become green). (4) After replication, BA2001 is released and infects all presynaptic neurons that are connected to the Ad-Cre infected projection neurons (presynaptic neurons are now green). These presynaptic neurons include interneurons in the spinal cord, the dorsal root ganglia (DRG) and supraspinal neurons that project to the cord.

Fig 1 briefly schematizes the strategy, in this case using the Cre-dependent pseudorabies virus vector, BA2001 [1]. First, we inject a Cre-expressing adenovirus (Ad-Cre) in a presumptive target of the PNs of interest (1). This injection results in uptake of Ad-Cre by the neurons at the injection site (area A) but importantly, also by terminals of all PNs that target area A. Retrograde transport of the Ad-Cre then initiates Cre expression in the cell bodies of all infected PNs (for example, in area B; neurons become blue in 2). Second, we inject BA2001 in area B, which results in infection of most, if not all projection neurons (and interneurons) in the region of the retrograde labeling. However, because replication of BA2001 is Cre-dependent, this virus will only replicate in neurons that express Cre-recombinase (blue neurons in 2), i.e., in Ad-Cre-infected PNs retrogradely labeled from area A (blue neurons in which BA2001 replicates are denoted by green color in 3). Finally, after replication in the Cre-expressing PNs, the BA2001 vector is released and infects neurons that are presynaptic to the Cre-expressing PNs. Importantly when the BA2001 viral vector is rendered competent, there is co-expression of a GFP reporter gene in the Cre-expressing cells. The consequent fluorescent labeling is easily detectable and importantly restricted to the Cre-expressing PNs and to its presynaptic inputs (presynaptic neurons “become” green in 4). Below we illustrate examples in which the strategy is adopted in different CNS regions.

### Adenovirus-Mediated Cre Expression in Projection Neurons of the Mouse CNS

Our laboratory is particularly interested in circuits that underlie the transmission of pain messages. For this reason, our first studies examined presynaptic inputs to spinal cord projection neurons at the origin of two pain-relevant systems: one that targets the pontine parabrachial nucleus (PB), a major relay through which painful inputs engage limbic structures and another, the ventrobasal thalamus (VB), a relay in the spinothalamic projection that terminates in the somatosensory cortex and is more associated with the sensory discriminative properties of pain messages. The first step in these studies is to inject stereotaxically the Ad-Cre into the PB or VB of wild-type mice. Ideally one would map the Cre-expressing projection neurons with an antibody directed against the Cre protein. In our experience, however, existing antibodies only detect the enzyme at the injection site, presumably because there are only very low levels of Cre in the Ad-Cre retrogradely infected neurons.

**Fig 2:**
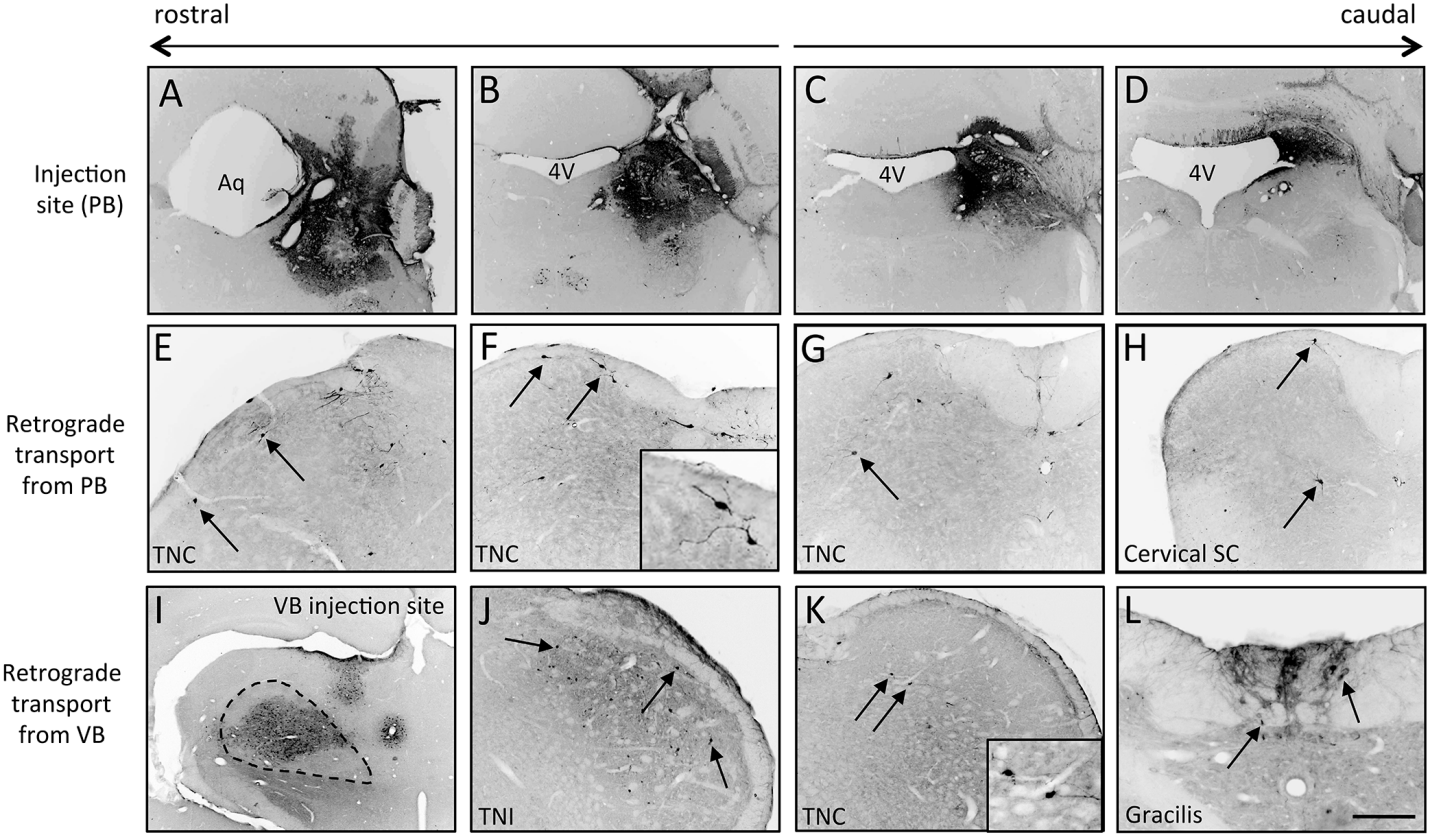
Targeting expression of Cre to projection neurons using retrogradely transported adenoviral vectors. Injection of Ad-Cre in the parabrachial nucleus (A-D) of a GFP-reporter mouse results in expression of GFP in many PNs of the trigeminal nucleus caudalis (arrows in E-G; magnified in inset) and cervical spinal cord (arrows in H). (I-L) Injection of Ad-Cre in the ventrobasal thalamus (VB; I) of a GFP-reporter mouse also results in expression of GFP in TNC projection neurons (arrows in J, K; magnified in inset) as well as in neurons of the nucleus gracilis (arrows in L). Aq: aqueduct; 4V: 4^th^ ventricle. Scale bar is 300μm in A-D and I; 150μm in E-H and J-L.

To address this limitation we also injected Ad-Cre in mice in which expression of yellow fluorescent protein (YFP) is induced after Cre recombination. In these Ad-Cre infected mice, YFP-expressing neurons correspond to Cre-expressing cells, i.e., Ad-infected neurons. Fig 2 shows that 7 days after Ad-Cre injection in the PB, we not only recorded YFP staining at the injection site (Fig 2A–2D) but also in several CNS neuron populations known to project directly to the PB [5], including neurons in lamina I of the trigeminal nucleus caudalis (TNC; Fig 2E–2G) and in the dorsal horn at cervical spinal segments (Fig 2H). Similarly, injections of Ad-Cre in the VB not only produced YFP+ neuron labeling near the injection site (Fig 2I), but also in the trigeminal nuclei interpolaris (TNI; Fig 2J) and caudalis (Fig 2K) as well as in the dorsal column nuclei (DCN; Fig 2L), each of which is known to contain PNs that target VB. These discrete labeling patterns confirm that there is terminal uptake of the Ad-Cre and its subsequent retrograde transport by PNs that target the injection site.

### Retrograde Transneuronal Transfer of BA2001 Reveals the Inputs to Projection Neurons in the Mouse Trigeminal Nucleus Caudalis

**Fig 3:**
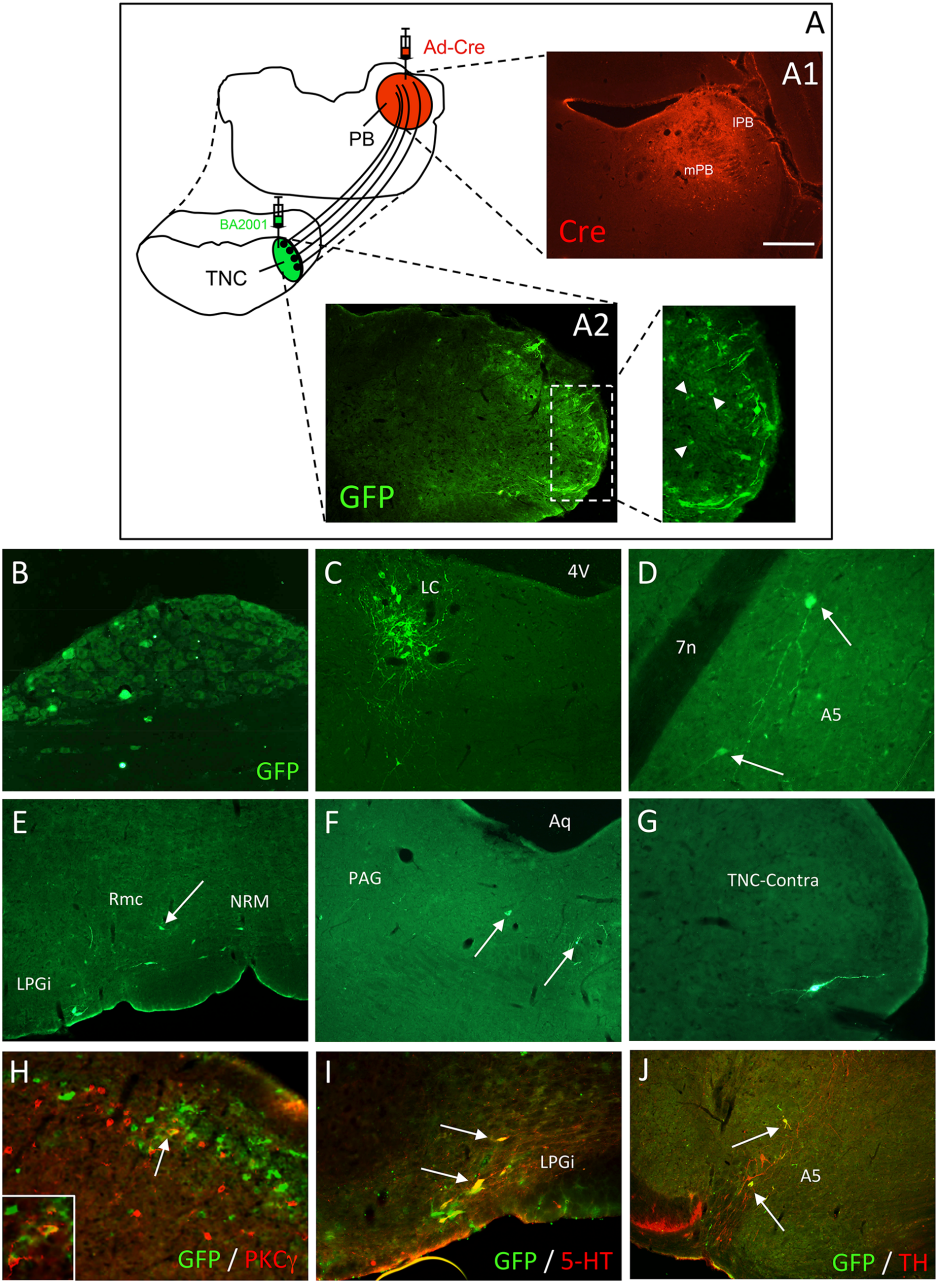
Inputs to projection neurons of the mouse trigeminal nucleus caudalis (TNC). (A) Injection (red) of Ad-Cre in the parabrachial nucleus (PB) infects neurons at the injection site (inset shows Cre-immunoreactivity; red in A1), where there is uptake by terminals of any projection neurons that terminate in the PB, including a subpopulation of TNC neurons. Subsequent injection of BA2001 (green) in the TNC infects all neurons at this injection site, but replication of BA2001, manifest by GFP expression (green in A2), only occurs in Cre-expressing TNC projection neurons (i.e., Ad-Cre retrogradely infected). Inset shows several GFP-expressing TNC neurons (arrowheads). (B-G) Once BA2001 becomes replication competent, it is retrogradely transferred to subpopulations of neurons that are presynaptic to the Ad-Cre-expressing PNs. These presynaptic neurons included sensory neurons of the trigeminal ganglia (B) and neurons of the locus coeruleus (LC; C), A5 (D), nucleus raphe magnus (NRM; E), periaqueductal grey (PAG; F) and even the contralateral TNC (G). Arrows point to examples of GFP-positive neurons. (H-J) Double labeling reveals that presumptive excitatory interneurons that express PKCγ (red in H), serotonin (5HT)-expressing neurons of the medullary lateral paragigantocelularis (LPGi; red in I) and tyrosine hydroxylase (TH)-expressing neurons of A5 (red in J) are among the neurons that are presynaptic to PNs of the TNC. See also S1 Fig. 4V: 4^th^ ventricle; 7n: 7^th^ nerve; Aq: Aqueduct; Rmc: nucleus reticularis magnocellularis. Scale bar: 300μm in A1; 150μm in A2 and B; 100μm in C and E-G; 50μm in D and H-J.

The preceding results establish the feasibility of targeting expression of Cre to subpopulations of PNs using a retrograde Ad-Cre viral tracer [6]. Next, we asked whether the Ad-mediated expression of Cre in PNs is sufficient to restore the replication competency of the Cre-dependent viral vectors so as to allow their subsequent transneuronal transfer. Here, we injected wild type mice with Ad-Cre in the PB and at the same time, the Cre-dependent transneuronal retrograde tracer, BA2001, in the TNC. Two days after these injections, we detected GFP+ neurons in superficial laminae of the TNC (Fig 3), which indicates that a Cre recombination event had indeed occurred, one that rendered the BA2001 competent. Importantly, we rarely detected GFP+ neurons in mice that were not injected with the Ad-Cre (data not shown), which confirms that the GFP labeling in co-infected animals was the result of the Cre recombination event, rather than from a leak of the transcriptional cassette inserted in BA2001.

In these double-injected animals, we not only detected GFP-expressing neurons in lamina I of the TNC, but also in deeper laminae (Fig 3A arrowheads in inset). Notably, we could immunostain many GFP-positive neurons for the gamma isoform of protein kinase C (PKCγ, a marker of excitatory interneurons of inner lamina II (Fig 3H). Because the latter interneurons do not project to the PB (and thus do not express Cre), we conclude that BA2001 was transneuronally transferred, retrogradely, from the Ad-Cre infected PNs of lamina I to interneurons of lamina II. However, because of the very rapid multisynaptic transport of BA2011, we cannot determine whether the PKCγ labeling represents a monosynaptic or polysynaptic input to the PNs.

In addition to the TNC, we detected GFP+ neurons in other areas known to send projections to the TNC. Notable among the latter was the trigeminal ganglion (Fig 3B), which contains the cell bodies of sensory neurons that terminate in the TNC. Other labeled neurons were recorded in various brainstem nuclei, including the catecholaminergic (TH+) locus coeruleus (LC, Fig 3C and S1 Fig), A5 (Fig 3D, 3J and S1 Fig) and A7 (data not shown) cell groups, the serotonergic (5HT+) nucleus raphe magnus (NRM, Fig 3E and S1 Fig) and the lateral paragigantocellularis nucleus (LPGi, Fig 3E, 3I and S1 Fig) and the nucleus reticularis magnocellularis (Rmc; arrow in 3E) of the rostral medulla, the periaqueductal gray (PAG: Fig 3F) and the nucleus praepositus hypoglossi (data not shown). Interestingly, we also detected GFP+ neurons in the contralateral TNC (Fig 3G), which is consistent with reports of bilateral crosstalk among neurons of TNC. Taken together, our results demonstrate that Ad-mediated expression of Cre in subpopulations of PNs of the TNC can restore the replication competency of BA2001, with a concomitant expression of GFP, and subsequent transneuronal retrograde transfer of the BA2001. The pattern of labeling reveals a diverse and widespread population of neuronal networks that influence parabrachial projecting neurons of the TNC.

### Inputs to Mouse Parabrachial Nucleus Neurons that Project to the Amygdala

**Fig 4:**
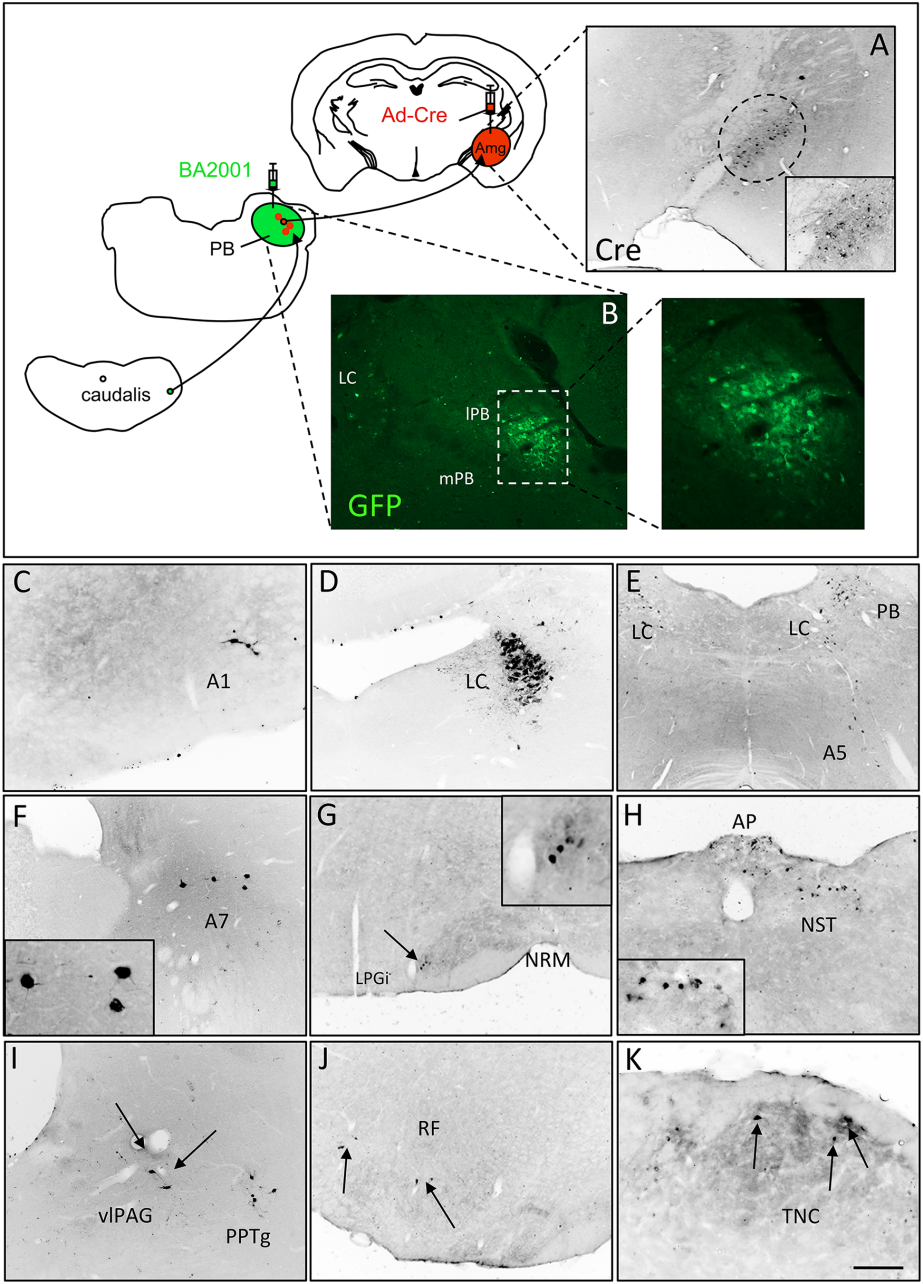
Presynaptic inputs to projection neurons of the mouse parabrachial nucleus (PB). Injection of Ad-Cre in the amygdala (Amg) and BA2001 in the PB results in expression of Cre in Amg neurons (black, A) and GFP in projection neurons of the PB that terminate in the Amg (green, B). Subsequent retrograde transfer of BA2001 labels a subpopulation of neurons that are presynaptic to PB projection neurons. The inputs derive from catecholaminergic neurons of the medullary A1 (C), locus coeruleus (LC; D), A5 (E) and A7 (F), as well as neurons in the lateral paragigantocellularis (LPGi; G), area postrema (AP; H), nucleus of the solitary tract (NST; H), ventrolateral PAG (vlPAG; I), pedunculopontine tegmentum (PPTg; I), scattered neurons in the reticular formation (RF; J) and neurons in superficial laminae of the TNC (K). Scale bar: 300μm in A; 100μm in B and 150μm in C-K.

In these studies we examined circuits that underlie the engagement of affective/emotional circuits by painful stimulation. Previous studies demonstrated that the parabrachial nucleus is a critical relay between spinal cord nociceptive circuits and the amygdala, a limbic system locus that engages emotional circuits of the brain [5, 7–9]. However, in these earlier studies, the connections from the spinal cord to the amygdala were examined in separate experiments (viz., spinal cord to PB and PB to amygdala). Here, we co-injected mice with Ad-Cre in the amygdala (Amg; Fig 4A) and BA2001 in the PB (Fig 4B). As expected, after two days of transport, we detected large numbers of GFP+ neurons in the ipsilateral PB. But now in a single experiment, and in agreement with other studies [5, 7, 10–12], we also detected neurons presynaptic to PB projection neurons. The inputs originated from several brainstem areas, including most noradrenergic cell groups (Fig 4C–4F), the lateral paragigantocelluar nucleus (LPGi; Fig 4G), the area postrema (AP; Fig 4H), the nucleus of the solitary tract (NST; Fig 4H), the ventrolateral periaqueductal gray (vlPAG; Fig 4I), the pedunculopontine tegmental nucleus (PPTg; Fig 4I), in scattered neurons of the medullary and pontine reticular formation (RF, Fig 4J) and in laminae I and II of the TNC (Fig 4K). These results demonstrate that the dual Ad-Cre/BA2001 injection can reveal both direct and indirect, presynaptic inputs to subpopulations of PNs in the parabrachial nucleus.

### Inputs to Projection Neurons of the Mouse Lateral Geniculate Nucleus (LGN)

**Fig 5:**
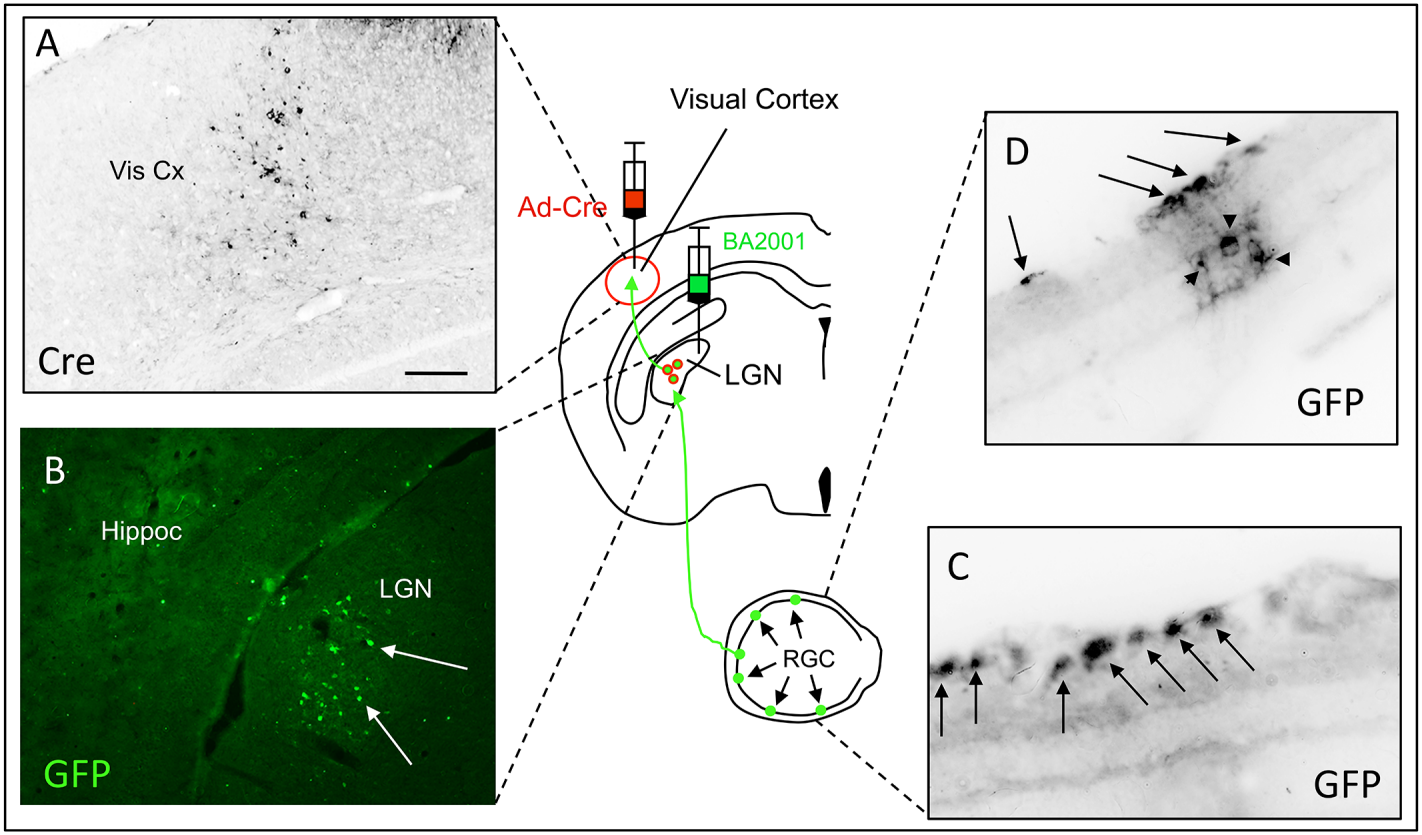
Presynaptic inputs to projection neurons of the mouse lateral geniculate nucleus (LGN). Injection of Ad-Cre in the visual cortex (Vis Cx) and BA2001 in the ipsilateral LGN results in expression of Cre in cortical neurons (black, A) and GFP in projection neurons of the LGN (green, B) that terminate in the visual cortex. Subsequent retrograde transfer of the BA2001 labels a subpopulation of neurons that are presynaptic to these LGN projection neurons, including retinal ganglion cells (RGC; arrows in C, D) as well as presumed bipolar cells (arrowheads in D) that target the RGC layer. Hippoc: hippocampus. Scale bar: 100μm in A-B and 50μm in C-D.

Finally, to establish the broad utility of this dual viral tracing system we turned to the visual system, where connections of major projection systems are well established. Here we co-injected mice with Ad-Cre in the visual cortex (Vis Cx; Fig 5A), to label thalamocortical projections from the LGN, and with BA2001 directly into the LGN (Fig 5B). As expected, we detected many GFP+ neurons in the geniculate, confirming retrograde transfer of the Ad-Cre from the cortex to the LGN, but we also recorded extensive labeling of retinal ganglion cells (RGC; arrows in Fig 5C and 5D) and presumptive bipolar cells (arrowheads in Fig 5D), which target the RG cell layer. Consistent with other studies that used more traditional tracing methods [13], we also observed retrograde transneuronal labeling of neurons in the hypothalamus (data not shown). Taken together, these results demonstrate that this dual Ad-Cre/BA2001 injection can reveal both direct and indirect, presynaptic inputs to subpopulations of PNs in diverse circuits of the mouse CNS.

### Inputs to Projection Neurons in the Rat

**Fig 6:**
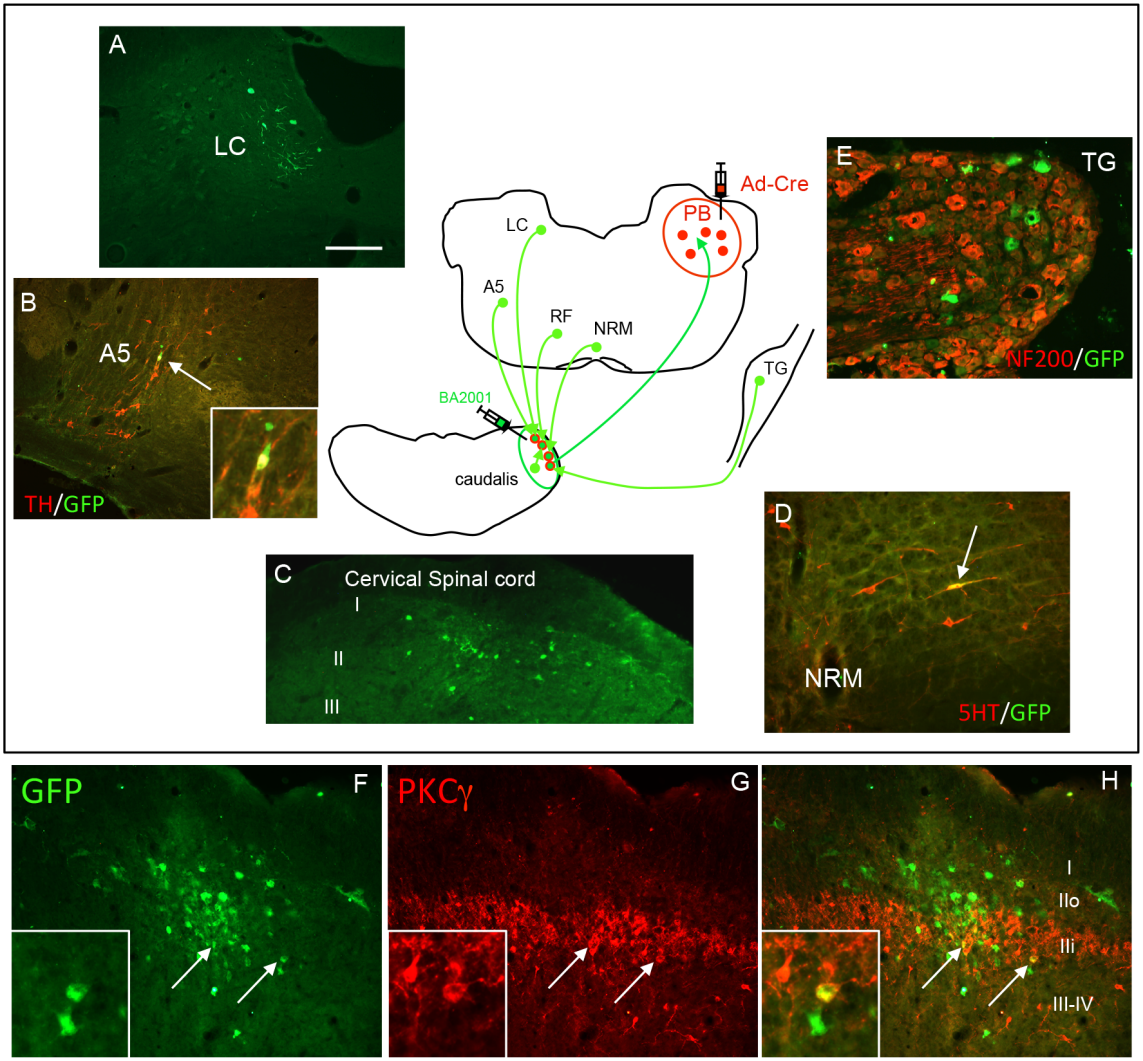
Dual viral infection in the rat. As in the mouse, co-injection of Ad-Cre in the PB of the rat and BA2001 in the TNC resulted in retrograde transneuronal transfer of BA2001 from TNC projection neurons to various brainstem loci, including TH-expressing neurons of the locus coeruleus (LC; A) and A5 (B), neurons in the superficial laminae of the cervical spinal cord (C), 5HT-expressing neurons of the nucleus raphe magnus (NRM; D) and sensory neurons of the trigeminal ganglion (TG; E). Double labeling with NF200 (red in E), which identifies sensory neurons with myelinated axons, reveals that the majority of retrogradely-infected BA2001 neurons of the TG is unmyelinated (green in E). (F-H) Double labeling demonstrated that some interneurons presynaptic to the projection neurons express PKCγ (red in G,H). Insets show high magnification of double-labeled neurons. Scale bar: 100μm.

As noted above, a great advantage of this new approach is that it does not depend on the availability of Cre-expressing mouse lines, but rather can be adapted for studies in a variety of animals. The only requirement is that the viruses transport in the particular animal and that the virus can be rendered competent. Here we turned again to the trigemino-parabrachial circuit, but now in the rat. We made injections of Ad-Cre in the PB and of BA2001 in the TNC and, as in the mouse, we recorded extensive neuronal GFP labeling in both projection neurons and interneurons of the TNC. In part because of the larger tissue and cell size, it was easier to interpret results from studies in which we combined immunocytochemical labeling of neurochemical markers of the retrogradely labeled neurons. Fig 6, for example, shows PKCγ-expressing interneurons of inner lamina II (Fig 6F–6H) transduced by BA2001. Fig 6A–6E illustrate other examples of neurons that either directly or indirectly input TNC projection neurons that target the PB, including GFP+ primary afferent neurons in the trigeminal ganglia and in various brainstem loci that are likely at the origin of descending controls that influence projection neurons (directly and indirectly). For example, as in the mouse we recorded large number of brainstem catecholaminergic (Fig 6B) and serotonergic (Fig 6D) cell groups that target lamina I projection neurons.

### Monosynaptic Inputs to Projection Neurons of the TNC

Despite the advantages inherent in the combined Ad-Cre/BA2001 transneuronal tracing system there are some limitations. For example, because of the rapid transport of BA2001, it can be difficult to differentiate between direct (i.e., monosynaptic) and indirect (i.e., polysynaptic) inputs to the primary, Cre-expressing neurons. In fact, we previously reported that both direct and indirect inputs are detectable within 24h of the virus infection [2]. Conceivably, shorter survival times could limit the BA2001 spread to neurons that are immediately presynaptic to the projection neurons, but variations in transport times will inevitably make interpretation of such results difficult. To overcome this limitation, here we introduced the monosynaptic rabies viral system, in which only first order presynaptic inputs are revealed [3]. Neurons that are labeled with BA2001 that also appear when rabies is used, in separate experiments, almost certainly correspond to those immediately presynaptic to the Cre-expressing PNs, i.e., they are monosynaptically connected to the PNs.

**Fig 7:**
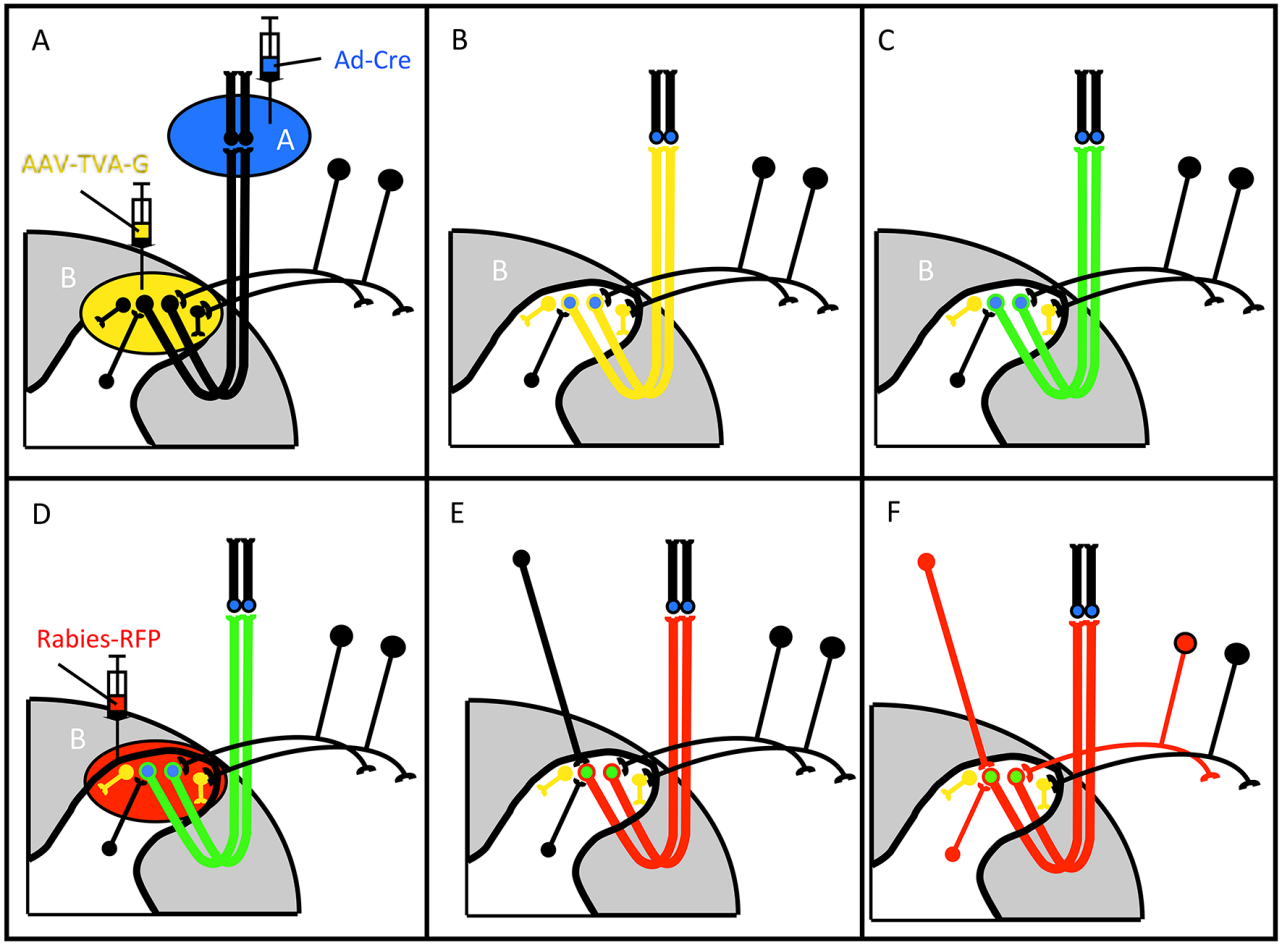
Dual viral infection strategy for the study of monosynaptic inputs to subpopulations of projection neurons. (A-B) There are common features to the strategy that uses the monosynaptic rabies system and BA2001 (see Fig 1). Co-injection of Ad-Cre in a CNS target (Area A, blue) and an AAV that expresses a Cre-dependent TVA receptor and G-protein (AAV-TVA-G) in area B (yellow) initiates expression of Cre (blue neurons in B) and TVA-G (yellow neurons in B) in projection neurons of area B that terminate in area A. (C) Because AAV-TVA-G also expresses GFP upon Cre-mediated recombination, the AAV-infected projection neurons also express GFP (green neurons in C). Injection of rabies-RFP in area B (D) results in infection only of the TVA-expressing projection neurons (red neurons in E) and because of G-expression, subsequent retrograde transfer of the rabies to neurons that are immediately presynaptic to the projection neurons (red neurons in F).

Fig 7 illustrates the strategy involving injection of the rabies virus. This virus requires expression of the avian receptor TVA, for its uptake into the neuron(s) of interest; transneuronal retrograde transfer from the projection neuron to presynaptic neurons requires expression of the glycoprotein G. Therefore, in addition to providing Ad-Cre via retrograde transport into the projection neurons, in these studies we made a local injection of a Cre-dependent AAV-TVA-G viral vector, which provides *in trans*, TVA and G, to the projection neurons. Because viral expression of TVA and G, as well as a GFP reporter, are Cre-dependent, the rabies can only “enter” Ad- and AAV-double-infected neurons. To ensure adequate TVA and G expression, we waited two weeks before injecting the rabies. To follow the spread of the rabies virus we monitored the red fluorescent protein (RFP) that the rabies virus expresses.

**Fig 8:**
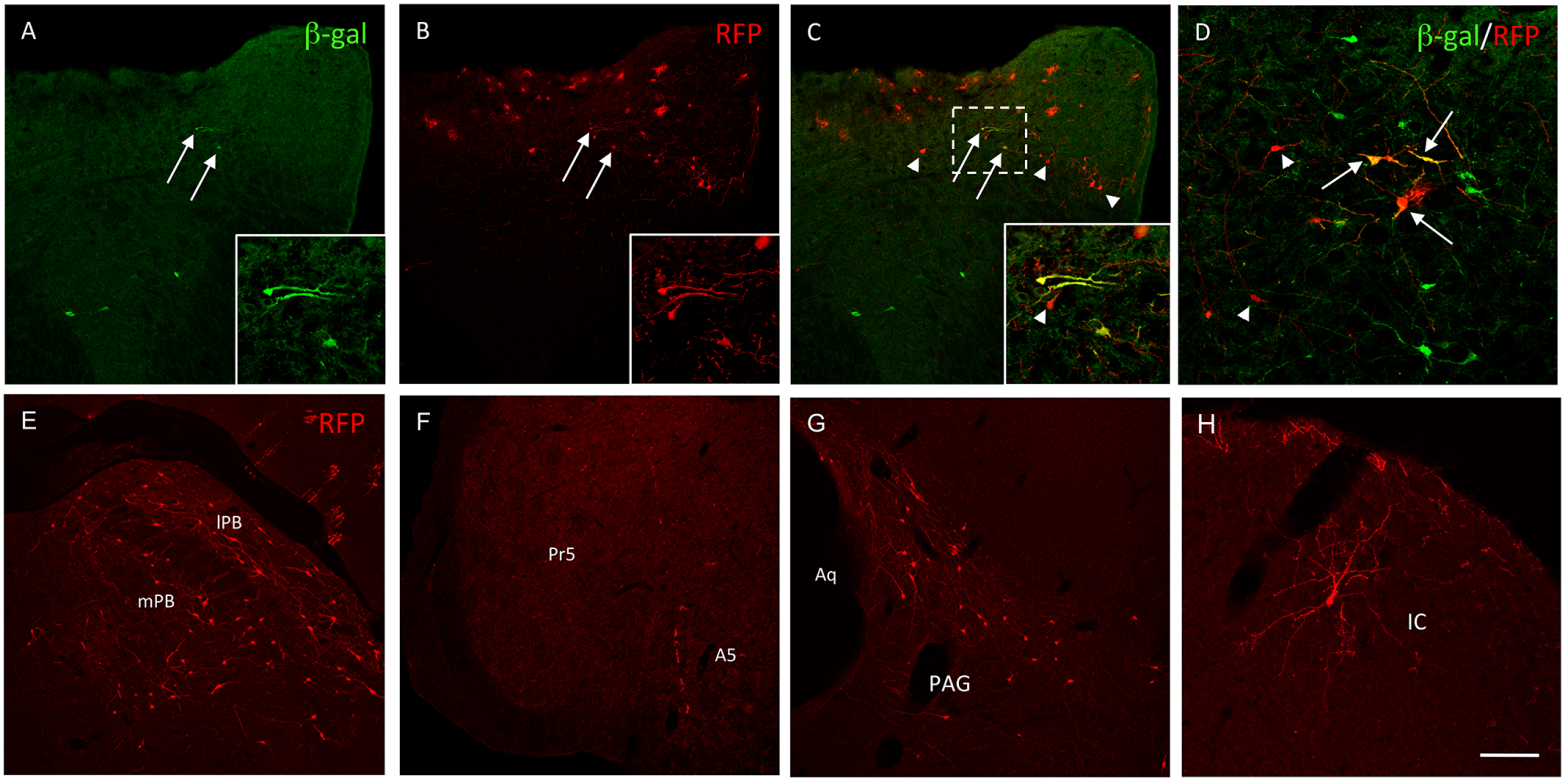
Rabies-based dual viral injection reveals monosynaptic inputs to projection neurons. (A) Injection of Ad-Cre in the PB of LacZ-reporter mice results in expression of ß-galactosidase (ß-gal, green) in projection neurons of the TNC. (B) Injection of the AAV-TVA-G in the TNC, followed 2 weeks later by injection of rabies-RFP results in RFP expression (red) in many neurons of the TNC. (C-D) Some RFP+ neurons (i.e. rabies-infected) also express ß-gal (arrows; magnified in insets). These RFP+/ß-gal+ neurons correspond to 1^st^ order projection neurons that terminate in the PB. Single labeled RFP+ neurons (arrowheads in D and inset in C) correspond to neurons that are immediately presynaptic to TNC projection neurons that target the PB. (E) Co-injection of Ad-Cre in the amygdala and AAV-TVA-G in the PB, followed 2 weeks later by a rabies injection in the PB, results in expression of RFP in many neurons of the PB (red). (F-H) Subsequent retrograde transfer of the rabies to presynaptic neurons reveals the neuronal networks that provide monosynaptic inputs to PB neurons that project to the amygdala. These inputs originate from neurons in A5 (F), the PAG (G) as well as scattered neurons in the inferior colliculus (IC; H). The rabies RFP labeling can occasionally reveal extensive dendritic arborization of the presynaptic neurons (H). Pr5: Principal 5; Aq: Aqueduct. Scale bar: 100μm in A-C and E-H and 75μm in D.

Our first studies again examined the trigemino-parabrachial circuit. First, we co-injected mice with Ad-Cre in the parabrachial nucleus and AAV-TVA-G in the trigeminal nucleus caudalis. Two weeks later we injected the rabies virus in the TNC. To address a complication that results from difficulty in identifying the Cre-expressing neurons (GFP expression is largely inhibited by the rabies; [3]), we performed our initial experiments in the Rosa26-LacZ reporter mouse [14], in which LacZ is Cre-dependently expressed. In these mice, immunocytochemical localization of ß-galactosidase (ß-gal), the protein product of *lacZ*, provided an independent reporter for the projection neurons that express Cre. Fig 8 illustrates that the combination of Ad-Cre/AAV-TVA-G with rabies can indeed reveal presynaptic inputs to subpopulations of PNs of lamina I of the TNC. We not only detected ß-gal+/RFP+ double-labeled neurons in the TNC, which correspond to the Ad-Cre infected 1^st^-order neurons in which replication of rabies is restored (arrows in Fig 8A–8D), but also many RFP+ single-labeled neurons in adjacent laminae of the TNC (arrowheads in Fig 8C and 8D). These RFP+, but l*acZ*-negative neurons correspond to neurons that are presynaptic to the RFP+/*lacZ*+ projection neurons of the TNC. In other words, the rabies vector is not only functional but its retrograde transport occurs even when three viruses (Ad, AAV and rabies) infect the projection neuron.

### Monosynaptic Inputs to Projection Neurons of the Parabrachial Nucleus

In the next experiments, we analyzed the pattern of rabies virus labeling following injection of Ad-Cre in the amygdala and AAV-TVA-G/rabies in the PB (Fig 8E–8H). As expected, here we not only detected many RFP+ (i.e. rabies-infected) neurons at the injection site (PB; Fig 8E) but also at some distance from the Cre-expressing projection neurons. For example, we recorded RFP+ neurons in A5 (Fig 8F), in the lateral and ventrolateral PAG (Fig 8G) and even a few cells in the dorsal part of the inferior colliculus (Fig 8H). These observations agree with some earlier reports [15, 16], but now we can conclude that these latter regions make direct (i.e., monosynaptic) connections with PB neurons that project to the amygdala.

Compared to BA2001, however, the rabies infections resulted in fewer labeled areas. For example, we rarely detected labeled neurons in the NST, PPTg or TNC following PB injections with rabies. As many of the regions identified with BA2001 have been shown to project directly to the PB (e.g., the NST), we do not believe that the more extensive pattern observed after BA2001 injection reflects its multisynaptic retrograde transport. Transneuronal labeling of BA2001 undoubtedly occurs, however, the rather limited rabies virus retrograde labeling of neurons distant from the Cre-expressing projection neurons suggests that there may be an as yet unexplained limitation to the uptake and transfer of this strain of rabies virus.

**Fig 9:**
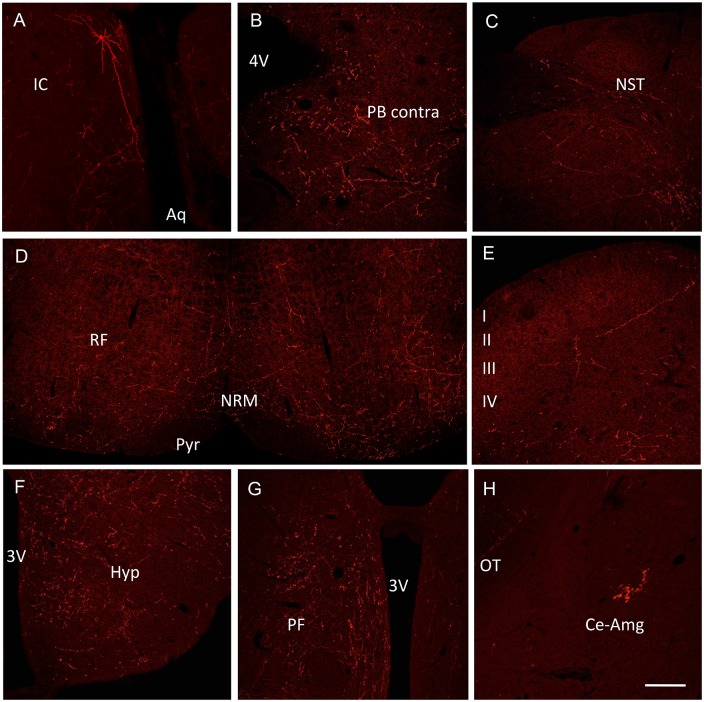
Extensive rabies-RFP labeling of the axonal arbors of projection neurons. (A-H) Because of the very strong rabies-driven RFP signal, there is extensive labeling of both the primary projection neurons and their immediately presynaptic neuronal inputs, including significant dendritic (A) and axonal arbors (B-H). Aq: aqueduct; IC: inferior colliculus; 4V: 4^th^ ventricle; PB contra: contralateral parabrachial nucleus; NST: nucleus of the solitary tract; RF: medullary reticular formation; NRM: nucleus raphe magnus; Pyr: pyramids; 3V: 3^rd^ ventricle; Hyp: hypothalamus; PF: parafascicular thalamus; OT: optic tract; Ce-Amg: central nucleus of the amygdala. Scale bar: 50μm.

Despite the limited number of labeled presynaptic neurons, there is one particularly remarkable feature of the rabies-labeled neurons. Because the rabies-driven RFP signal is very strong, we found that the labeling of both the primary PN and of neurons presynaptic to the PNs was exceptional, often Golgi-like, with extensive filling of dendrites and widespread axonal arborization (e.g. Figs 8H and 9). This property of rabies-infected neurons was particularly valuable in animals in which we did not detect RFP+ cells beyond the injection site. For example, in animals in which PB injections of rabies only revealed RFP+ neurons in the PB, we nevertheless observed RFP+ axon collaterals that targeted the contralateral PB (Fig 9B), the NST (Fig 9C), throughout the reticular formation (Fig 9D) and regions as distant as the cervical spinal cord (Fig 9E). In the brain, we recorded parabrachial collaterals in the hypothalamus (Fig 9F), parafascicular nucleus of the thalamus (Fig 9G) and in the amygdala (Fig 9H). Although it is possible that these anterogradely labeled axonal arbors derived from interneurons or Cre-negative projection neurons that are presynaptic to the Cre-expressing projection neurons within the PB, we favor the view that the most extensively labeled neurons were primary projection neurons that target the amygdala.

### Combination of Rabies Vectors and TVA-G-Expressing Transgenic Mice

Although valuable, the viral approach using the rabies system can be technically challenging (see Discussion), as it requires simultaneous infection of neurons by 3 viral vectors. For this reason, the probability of having neurons co-infected with 3 viruses is small, which reduces subsequent detection of retrograde transfer of the vector. To overcome this limitation, we also performed our dual infection approach in transgenic mice that Cre-dependently express TVA and the G protein [17]. The approach obviates the need for the AAV-TVA-G. Rather, in these experiments, we injected a GFP-expressing rabies vector into the TNC two weeks after PB injection of a Cre- and RFP-expressing adenoviral vector. The latter virus targets expression of Cre and RFP to the TNC projection neurons.

**Fig 10:**
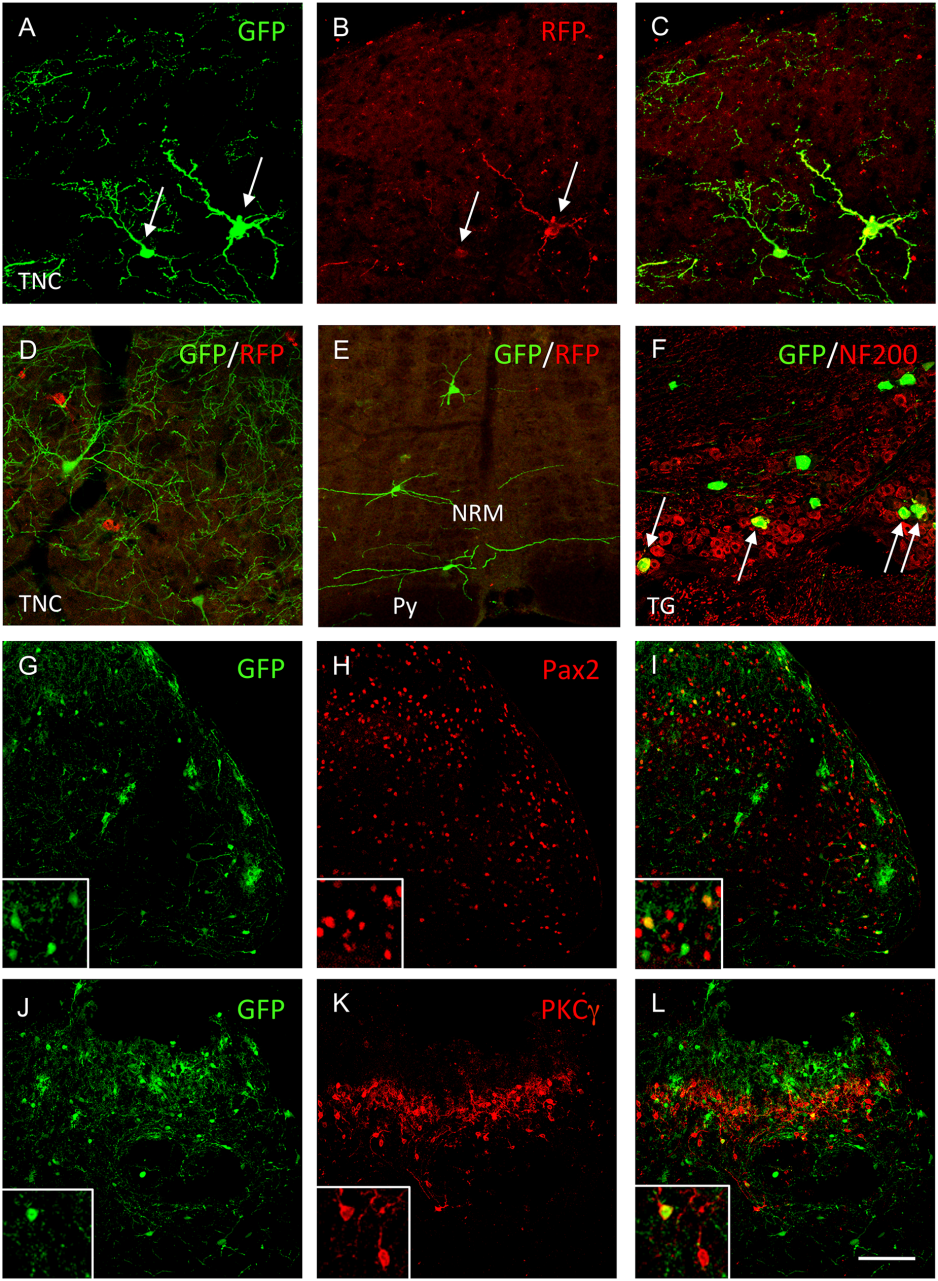
Dual viral injection in transgenic mice that Cre-dependently express TVA-G also reveals monosynaptic inputs to projection neurons. (A-C) Injection of Ad-Cre-RFP (red) in the PB of transgenic mice that ubiquitously and Cre-dependently express the TVA and G-proteins, allows subsequent infection of TNC projection neurons by the rabies-GFP (green). Arrows in A, B point to double-labeled GFP-RFP projection neurons of the TNC. (D-F) Retrograde transneuronal transfer of the rabies-GFP reveals subpopulations of neurons that provide monosynaptic inputs to these TNC projection neurons, including other neurons in superficial laminae of the TNC (D), in the NRM (E) and in a mixed population of TG neurons (F). Yellow labeling in F (arrows) points to NF200-GFP double-labeled neurons with myelinated axons. (G-L) Inhibitory interneurons that express Pax2 (red) and are presynaptic to the GFP-expressing projection neurons of the TNC are common (yellow in inset), but we only recorded isolated double-labeled PKCγ-expressing interneurons (red in K, L; yellow in inset). Scale bar: 75μm in F and 50μm in all other panels.

Fig 10 illustrates results from these experiments. Two weeks after injection of rabies into the TNC of the TVA-G-expressing transgenic mice, we detected GFP+ neurons in several laminae of the ipsilateral TNC. Some of the latter immunostained for RFP (Fig 10A–10C), which indicates that they were projection neurons, i.e., 1^st^ order neurons co-infected by rabies and Ad-Cre. We also recorded many single labeled GFP+ neurons throughout the brainstem (Fig 10D and 10E), as well as in a mixed population of trigeminal ganglion neurons (Fig 10F). These GFP+/RFP- neurons must correspond to neurons that are directly presynaptic to the PB-projecting neurons of the trigeminal nucleus caudalis. Among these presynaptic neurons were some in the TNC that we double–labeled for Pax2, a marker of inhibitory interneurons (Fig 10G–10I). Interestingly, we rarely found PKCγ-expressing interneurons (Fig 10J–10L) that co-labeled for GFP, indicating that these do not directly input the projection neurons.

## Discussion

Compared to more traditional approaches that use retrograde and anterograde tracers [18], our dual infection approach offers temporal and spatial control of the replication of the viral vectors. This feature permits the selective labeling of inputs to neurochemically-defined (Cre-expressing) subpopulations of PNs. In fact, by using an adenovirus [19, 20] to target Cre expression to distinct populations of PNs, we can control the neuronal population in which transport of the Cre-dependent viral tracers BA2001 and rabies are subsequently triggered. Most importantly, even though BA2001 is undoubtedly taken up by interneurons and many projection neurons at the injection site, it is *only* made competent in the PNs that express Cre, namely those that were retrogradely infected by the Ad-Cre. Of course, because the rabies requires the avian receptor TVA to enter cells as well as the viral glycoprotein G for their exit, control of the replication/transfer of the rabies virus is even tighter. The fact that the requisite expression of both TVA and G is Cre-dependent further limits the transneuronal spread of the rabies. And because the dual infection method does not require endogenous expression of Cre, this new approach is readily applied to larger animals, in which targeting discrete populations of projection neurons is likely less challenging than in the mouse. Finally, given the particular ability of a modified PRV (the so-called Brainbow-PRV) to distinguish 1^st^ order neurons (where viral replication is recovered) from others that are presynaptic, we believe that the versatility of our approach could be significantly enhanced by integrating it with this new vector [21,22].

Some limitations need to be addressed. For example, viral tracers often only label a subpopulation of neurons within a particular circuit. This limitation may arise because viral vectors, as for different retrograde tracers [23], do not have equal affinities for different neuronal populations [24]. It is also possible that variability in projection neuron arborization within a target region can restrict uptake and retrograde transport of the Ad-Cre to a subset of projection neurons. Somewhat related is the possibility that projection neurons with dense terminal fields are more easily infected than are neurons with sparse projections [25]. This variability in infection susceptibility, of course, makes quantitative analyses extremely difficult.

Another limitation relates to potential false positives. For example, in the PB-TNC projection system, it is possible that neurons that have collaterals terminating in the PB, where the Ad-Cre is injected, and in the TNC, where the BA2001 is injected, may be simultaneously infected by Ad-Cre and BA2001, after their retrograde transport. Such Ad/BA2001 double-infected PNs would undoubtedly express GFP, as these neurons would express Cre, and thus be mistakenly characterized as presynaptic to PNs of the TNC. A careful assessment of the individual pattern of retrograde labeling that occurs after a particular region is injected can reduce the incidence of these false positives. As a 3^rd^ viral vector (AAV-TVA-G) is used in the rabies studies, false positives may be avoided by injecting AAV vectors that only transport anterogradely [26, 27]. There are, in fact, serotypes that do not transport retrogradely (e.g., AAV2; [28]). Their use should prevent rabies infection of neurons that collateralize to the PB and TNC because the co-retrogradely labeled neurons would lack TVA. Furthermore, in cases where a neuron distant from the rabies/BA2001 injection site is double-labeled, i.e. Ad-Cre positive and rabies or PRV positive, one needs to be especially cautious as this double labeling will likely reflect a false positive. For this reason, when an unexpected connection is revealed, it is advisable to confirm the connection using more traditional anterograde tracing procedures.

Finally, because BA2001 is a transneuronal viral tracer that travels along polysynaptic pathways, there is a particularly difficult, but critical problem determining the order of infection in a multisynaptic circuit. The difficulty arises because of the very rapid (within 6h) replication cycle of the virus [29]. In fact, we detected both monosynaptic and polysynaptic connections within 24h of virus injection [2]. For this reason careful timing alone may not be adequate to distinguish primary from secondary neuronal labeling. On the other hand, here we show that by comparing the labeling patterns produced with BA2001 and rabies, it is possible to differentiate 2^nd^-order from higher-order neurons. For example, when we injected BA2001 and rabies in the PB (in separate animals), we detected retrograde transfer of both viruses to neurons of the PAG and the A5 cell group. We conclude, therefore, that BA2001 had labeled the same, immediately presynaptic populations of neurons that target PB neurons that project to the amygdala. On the other hand, BA2001, but not rabies, labeled many PKCγ-expressing interneurons, indicating that this subpopulation of excitatory interneurons likely targets projection neurons via polysynaptic circuits [30–32].

Cordero-Erausquin et al. [33] used a somewhat similar strategy to study the presynaptic inputs to lamina I projection neurons that terminate in the lateral PB. These authors followed transport of a PB-injected adenovirus encoding the fusion protein GFP-TTC (fragment C of the tetanus toxin) and recorded retrograde transfer of GFP-TTC into laminae I-II cells (including presumptive stalked cells) that were presynaptic to PNs of lamina I. However, in their approach, it is the GFP-TTC that is transferred, not the virus. As a result, there is dilution of the signal in the presynaptic cell, which may limit detection of very discrete projections. In contrast, in our approach once the competence of the viral vector is restored, there will be concomitant, strong expression of RFP/GFP. As a result our approach provides significant amplification of the signal in presynaptic neurons, regardless of input strength. For example, the robust virally-driven RFP signal in PNs allowed us to reveal exquisite morphological details of the cells of origin of the pathway under analysis, as well as their extensive collateralization throughout the brainstem. This advantage was valuable even in animals in which there was no retrograde transfer of rabies beyond the injection site (Fig 9).

### Dissecting Local TNC Circuitry Using the Dual Viral Infection Approach

Given our laboratory’s particular interest in pain circuitry, in a proof of concept set of experiments we tested the dual infection approach in previously documented CNS circuitry. In all cases, we found that Ad-mediated expression of Cre in PNs enabled replication of BA2001 and rabies in Cre-expressing neurons and subsequent transneuronal transfer of these viruses to presynaptic neurons. In general, our results were consistent with previous anatomical studies that used more traditional tracers [34–36]. As noted above, however, in contrast to traditional tracers, a clear advantage of the dual infection approach is that by comparing the patterns of spread of rabies and BA2001, it is possible to distinguish between mono- and polysynaptic inputs to specific populations of PNs. Furthermore, we can identify the neurochemistry of some of the presynaptic neurons. For example, we found that PNs of the TNC receive polysynaptic inputs from PKCγ-expressing excitatory interneurons of inner lamina II, but monosynaptic inputs from dorsal horn inhibitory (Pax2+) interneurons and from brainstem monoaminergic systems that target the TNC and spinal cord [34]. Taken together, our results highlight the complex ascending and descending neuronal networks that regulate projection neurons of the TNC and likely of the spinal cord [37] and offer an approach to dissecting these networks in greater detail. Finally, our demonstration of the utility of transneuronal retrograde transfer of BA2001 and rabies from projection neurons of the PB and LGN demonstrate that this novel viral tracing approach should be amenable for analysis of inputs to any CNS projection neurons. Importantly, because all the proteins necessary to transform BA2001 and rabies into replication-competent transneuronal tracers are provided *in trans* by the viral vectors, our approach should be feasible in many animals, in addition to Cre-expressing transgenic mice.

## Supporting Information

S1 Fig (Related to Fig 3): Inputs to projection neurons of the mouse trigeminal nucleus caudalis (TNC). Injection of Ad-Cre in the parabrachial nucleus and BA2001 in the TNC results in retrograde transfer of BA2001 to subpopulations of neurons that are presynaptic to the Ad-Cre-infected projection neurons of the TNC. These presynaptic neurons included tyrosine hydroxylase (TH)-expressing neurons of the locus coeruleus (LC; A-C) and A5 (D-F) as well as serotonin (5HT)-expressing neurons of the nucleus raphe magnus (NRM; G-I). Arrows point to examples of double labeled neurons and arrowheads to GFP+, single labeled neurons. Scale bar: 100μm.
(PDF)
