# Supplementary figures and images for: Presynaptic Inputs to Any CNS Projection Neuron Identified by Dual Recombinant Virus Infection
Source: PLoS One. 2015 Oct 15;10(10):e0140681. doi: 10.1371/journal.pone.0140681 (PMC4607402; doi:10.1371/journal.pone.0140681)

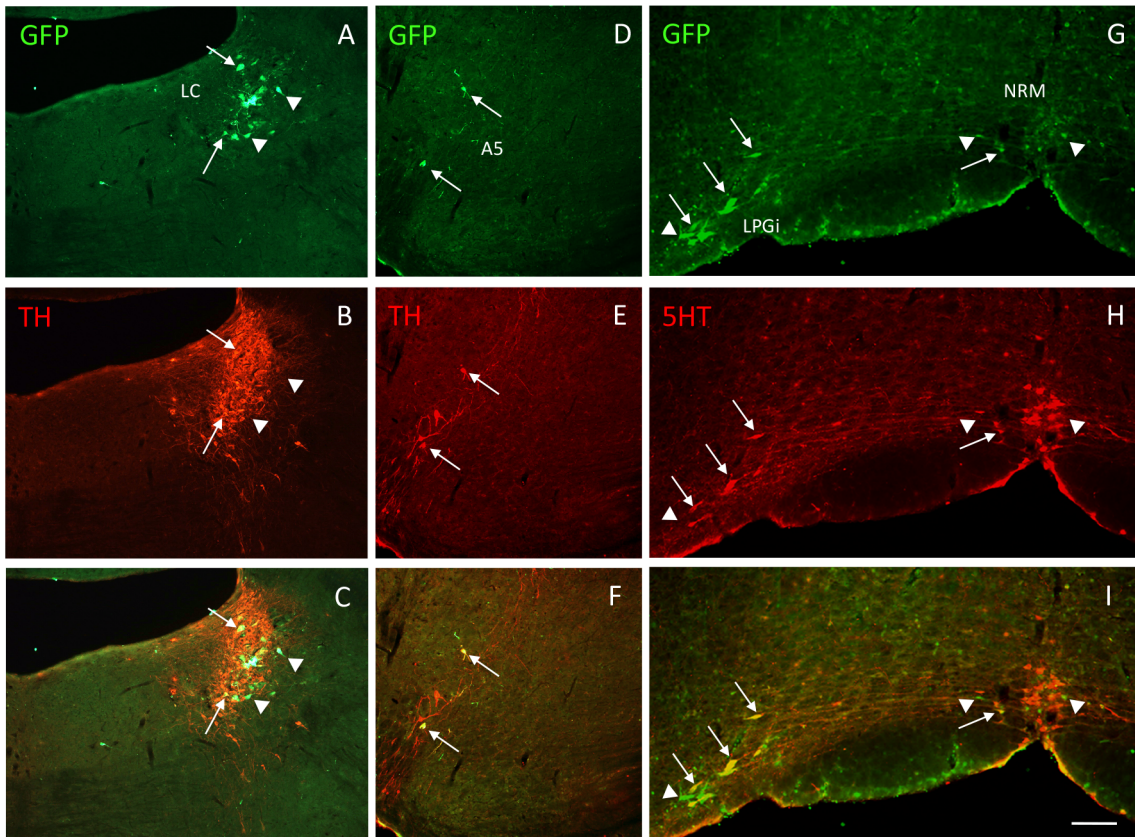

Supplement: S1 Fig — Injection of Ad-Cre in the parabrachial nucleus and BA2001 in the TNC results in retrograde transfer of BA2001 to subpopulations of neurons that are presynaptic to the Ad-Cre-infected projection neurons of the TNC. These presynaptic neurons included tyrosine hydroxylase (TH)-expressing neurons of the locus coeruleus (LC; A-C) and A5 (D-F) as well as serotonin (5HT)-expressing neurons of the nucleus raphe magnus (NRM; G-I). Arrows point to examples of double labeled neurons and arrowheads to GFP+, single labeled neurons. Scale bar: 100μm. (PDF) [file pone.0140681.s001.pdf]
